# The experience of professional nurses working with newly qualified nurses placed for community service in public health facilities in the City of Tshwane, South Africa

**DOI:** 10.4102/curationis.v44i1.2166

**Published:** 2021-05-24

**Authors:** Paulina D. Mabusela, Tendani S. Ramukumba

**Affiliations:** 1 Adelaide Tambo School of Nursing Science, Faculty of Science, Tshwane University of Technology, Tshwane, South Africa

**Keywords:** newly qualified nurses, professional nurses, community service, community service nurses

## Abstract

**Background:**

The newly qualified nurses (NQNs) were mandated to work for one year as community service nurses before being registered. During the placement, NQNs were supposed to be supervised and supported by professional nurses. On the contrary, professional nurses expected NQNs to be hands-on and provide quality care on completion of their training. Expectations of professional nurses created misperceptions regarding the objectives of community service. Therefore, exploring their experience would identify skills gap that is needed to be addressed.

**Objectives:**

This study explored and described the experiences of professional nurses working with NQNs placed for community service in the City of Tshwane.

**Method:**

A qualitative exploratory design was conducted. Individual interviews were carried out with 22 professional nurses in various public healthcare settings, such as a hospital, a community health centre and a clinic. Informed consent was obtained from all the participants and confidentiality and anonymity were maintained throughout the interviews. Creswell’s data analysis process was implemented.

**Results:**

Two themes emerged from this study, namely, experience of participants with NQNs and professional nurses. Participants experienced NQNs as not being competent to work independently and had to deal with unprofessional behaviour of NQNs. Participants supported NQNs, even though they were not empowered as mentors to NQNs.

**Conclusion:**

Participants were disappointed that NQNs were not competent and confident to work independently. They expressed their frustrations in behaviours displayed by NQNs. However, participants acknowledged NQNs’ individual differences and were supportive towards them even though they were not empowered for such responsibility.

## Introduction

Newly qualified nurses (NQNs) in South Africa, on completion of their training, as general, psychiatric and community nurses and midwives, according to *Regulation 425 of 22 February 1985* as amended (*Nursing Act, 33/2005:2*), are mandated to work for a period of one year (South African Nursing Council [SANC] 2007:76), before they can register as professionals. The programme is called community service and it is meant to capacitate NQNs (Department of Health 2013) and to provide an enabling environment for NQNs so that they can gain working experience and develop nursing care skills (Department of Health 2016:4). Rispel and Barron (2012:616) acknowledged the community service programme as an important development in recognising and managing the human resource crisis in the Department of Health.

South Africa’s healthcare system is predominantly nurse based; it is therefore important for nurses to be competent in order to meet the healthcare needs of the country. According to the Department of Health (2016:5), the main aim of the community service programme was ‘to ensure improved provision of health services to all citizens of the country.’ Therefore, community service should provide ‘young nurses with an opportunity to develop skill, acquire knowledge, behaviour patterns and critical thinking that will help them in their personal and professional growth’ (Department of Health 2016:5).

Whilst on community service placement, NQNs needed the support and encouragement of experienced professional nurses (Van Rooyen et al. 2018:35). Providing the needed support would ensure that NQNs acquire the necessary skills and effectively integrate the competencies they have acquired during their training. The support provided during community service can benefit the NQNs by building the much-needed confidence in their execution of duties.

Considering their experience and nursing skills, professional nurses were expected to work with and capacitate NQNs. Professional nurses were also expected to provide quality patient care, despite being faced with shortages of nursing personnel and material resources (Thopola, Kgole & Mamogobo 2013:175). Adding to the challenges of shortage of nursing personnel, professional nurses were expected to play the role of coaches and mentors to younger nurses. Similar circumstances are experienced internationally, as Phillips et al. (2013:107) cited that experienced professional nurses were expected to mentor new nurses whilst at the same time exposed to heavy workloads, excessive overtime and inflexible scheduling.

Most professional nurses saw NQNs as the answer to shortage of nursing personnel (Beyers 2013:39) and expected the presence of NQNs to bridge the shortage gap in health facilities. In their study, Netshisaulu and Maputle (2018:2) found that experienced midwives expected newly qualified midwives who were placed for community service to be hands-on immediately on arrival and to assume accountability and responsibility so that they could share the workload. However, experienced midwives were disappointed that newly qualified midwives were not work ready and could not practise independently soon after they qualified and graduated. Similar findings regarding incompetency of NQNs were shared by Nkoane (2015:68), who alluded to the fact that experienced professional nurses raised concerns that NQNs were not skilled enough to practise independently. Several researchers have attempted to identify and address the problems related to community service, such as the induction process (Makua 2016:281), mentoring of NQNs (Khunou & Rakhudu 2017:450) and lack of guidelines for implementation of community service.

Expectations of professional nurses demonstrated lack of understanding of the objectives of community service. Rispel and Barron (2012:616) also referred to the information gaps that still remained in the implementation of community service and the uncertainty about effective strategies and interventions to address the identified gaps. Considering the expectations of professional nurses from the NQNs, their experience in working with NQNs needed to be explored. Knowledge of professional nurses’ experience would help in identifying the gaps that can be addressed in developing guidelines for implementation of community service. To explore the experiences of professional nurses in working with NQNs, the research question developed was the following:

What are the experiences of professional nurses working with newly qualified nurses placed for community service in public health facilities in the City of Tshwane, South Africa?

The main study was aimed at developing guidelines for implementation of community service in the City of Tshwane. The experience of professional nurses working with NQNs placed for community service in public health facilities in the City of Tshwane was phase 2 of the main study. Phase 1 was a Systematic Literature Review, exploring the documented research on the experiences of newly qualified nurses placed for community service in South Africa. Therefore, the aim and objective of phase 2 were to explore and describe experiences of professional nurses working with NQNs placed for community service in public health facilities in the City of Tshwane, South Africa.

### Definition of concepts

#### Professional nurse

In South Africa, a professional nurse refers to a person who has been trained under R425 South African Nursing Council [SANC] 1985), has complied with the requirements of an Nursing Education Institution (NEI), which could be a nursing college for a nursing diploma or university for a nursing degree and is registered with the SANC.

For this study, a professional nurse is a nurse registered with the SANC, employed at a public health facility, at any level of healthcare delivery, who has worked directly with NQNs placed for community service.

#### Newly qualified nurses

Nurses who qualified under regulation R425 of the South African Nursing Council (SANC 1985) for comprehensive training in general, psychiatric and community nursing and midwifery and who are mandated to work for a period of 12 months in public health services before they can register with the SANC.

In the context of this study, an NQN means a person registered with the SANC in the category of community service nurse (*Nursing Act 2005:76*), who was placed for community service in the public health facilities.

#### Community service programme

Community service refers to the compulsory service that healthcare professionals are compelled to do in public health facilities, after they have received their academic qualifications. In this study, community service refers to the compulsory service placement that NQNs are mandated to do in public health facilities after they have graduated with their nursing diplomas or degrees, but before they are registered with the SANC.

## Research methods and design

The study was conducted at the institutions where professional nurses were employed and NQNs were placed for community service. A variety of settings were purposefully selected based on the placement of NQNs. The settings that were selected were a hospital, a community health centre and a clinic.

### Research design

A qualitative approach was used to explore and describe the experience of professional nurses working with NQNs who were placed for community service.

### Population and sampling

The accessible population consisted of all professional nurses who were employed in the selected public health facilities in the City of Tshwane, South Africa. Prospective participants should be willing to sign consent forms and qualify to participate based on the inclusion criteria. Professional nurses were included in the study if they had been registered with the SANC at the time of data collection, were working in the public health facilities and had been working with NQN for five years and more. Five years was regarded as sufficient time to have gathered relevant experience with the NQN and to be able to provide required data. Facility managers were excluded from the study because they were not working closely with NQN. To ensure in-depth and rich data, professional nurses who were information rich, based on their experiences of having worked with NQNs, and who were willing to provide the researcher with appropriate data were purposefully selected in the identified settings.

### Data collection

The study was contextual and the settings for data collection were the following public health facilities: a hospital, a community health centre and a clinic, in the City of Tshwane. As research involved an intrusion into the activities of an institution (Flick 2014:160), it was essential for the researcher to follow protocols to gain access and trust of institutions and participants. The procedure followed is explained under ethical considerations.

Subsequent to ethical considerations, permission was further pursued with the facility managers at the community health centre, and the clinics and from ward managers at the hospital to allow the researcher to access professional nurses for interviews. The background and objectives of the study were explained to professional nurses who met the inclusion criteria. Thereafter, an appointment for an interview was set with each potential participant.

An interview schedule was developed, guided by the research question and the research objective. The interview schedule was pre-tested on three professional nurses who met the inclusion criteria and gave informed consent. Two participants from whom the interview schedule was tested were from the hospital and one participant was from the clinic. Minor corrections were effected with regard to probing and validating. The responses for pre-testing the schedule were not included in the data analysis.

Verbal and signed informed consent for the face-to-face interview and to record the interview was obtained from professional nurses who volunteered to participate. Face-to-face semi-structured individual interviews were conducted. Each interview lasted between 25 min and 35 min. Participants responded to one main question, which was as follows: ‘could you please share with me your experiences in working with newly qualified nurses who are placed for community service in your facility?’ The main question was followed by probing and validating questions, which were directed by how the participants responded. Recorded interviews were copied on a disk and were password protected.

**FIGURE 1:**
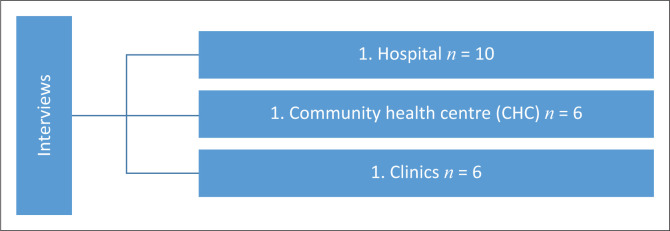
Number of participants in each facility.

As a result of shortage of nursing personnel, in the public healthcare facilities, the sample size was determined by the availability of professional nurses who met the set criteria; hence, six professional nurses were interviewed in the Community Health Care setting and another six in the clinic. At the community health centre, eight participants volunteered, but one declined just before the interview and one was disqualified as it was discovered at the start of the interview that she did not meet the inclusion criteria. At the hospital 10 professional nurses were interviewed. Saturation at the hospital was reached at the seventh participant and three more were interviewed to confirm the saturation. Figure 1 depicts the number of participants interviewed at each setting.

### Data analysis

Raw data were organised and converted into findings that answered the research question. The process of analysing qualitative data started with managing and organising raw data (Brink, Van der Walt & Van Rensburg 2018:180). In qualitative research, data analysis starts as the researcher collects data. The researcher started the analysis by taking field notes and analysing expressions such as tone of voice, facial expressions and body postures. Data analysis was carried out according to Creswell’s data analysis spiral (Cresswell 2014:182–188). The researcher applied the Creswell’s six steps approach to data analysis as described in Theron (2017:7). The researcher listened to the recorded data, transcribed and read it, organised the transcribed data, developed sub-themes and themes and then concluded the findings.

To conceal the professional nurses’ identity, the researcher coded their identity by first using the first letter(s) of their place of employment, followed by an ‘N’ (indicating ‘nurse’) and an allocated number to the participant. The coding was as follows: ‘H,N1’ would represent hospital nurse number one, ‘CHC,N2’ would represent community health centre nurse number two and ‘C,N3’ would represent clinic nurse number three.

### Trustworthiness

Trustworthiness is essential in qualitative research, and it was ensured through acknowledgement of four criteria, namely: credibility, dependability, transferability and confirmability. The researcher’s activities to achieve the criteria were as follows:

**Credibility:** It is achieved through prolonged engagement at the study site, by spending sufficient time building rapport and interacting with participants. Triangulation of data was done through the use of in-depth interviews with participants from different healthcare facilities. An independent quota was used to check the data against the emerged themes.

**Dependability:** a reliable, clear and concise research design was explained and implemented, thus it can be tracked from methodology to the results.

**Transferability:** Purposive sampling was applied and only professional nurses who were experienced in working with NQNs placed for community service were chosen to participate in the research. Inclusion and exclusion criteria were well set.

**Confirmability:** The researcher applied bracketing by identifying and setting aside any preconceived beliefs and opinions about the topic being researched. All accumulated evidence such as field notes, audio recordings and transcripts were kept safe for reference’s sake.

### Ethical considerations

Ethical approval to conduct the study was obtained from the Faculty of Science Committee for Research Ethics, in an institution where the study was supervised. Permission was obtained from the Department of Health and the Tshwane Research Ethics Committee. Written permission to collect data was sought from the chief executive officer of the hospital and the regional nursing managers of the community health centre and the clinic. The regional nursing managers guided the researcher on health facilities where NQNs were placed. This approach assisted the researcher to conveniently select relevant community health centre and clinic for data collection.

## Results

**FIGURE 2:**
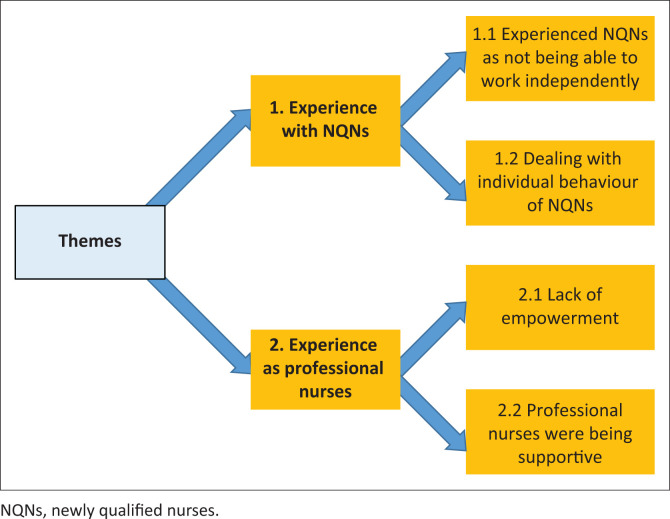
Themes and sub-themes for results.

Results of the interviews were grouped into themes. Two themes and four sub-themes emerged from the data analysis. Participants reported about their experiences with NQNs and reported about themselves. The identified themes are displayed in Figure 2.

The themes and sub-themes are discussed and substantiated with verbatim quotes from the participants.

### Theme 1: Experiences with newly qualified nurses

Participants’ experience in working with NQNs was that NQNs were not yet ready to practise independently and were not competent. They believed that NQN needed some intervention to improve their skills. They also experienced some of the NQN’s behaviours to be unethical.

#### Sub-theme 1.1: Experienced newly qualified nurses not being able to work independently

Participants experienced NQNs as still being dependent and still needed to be taught like they were accompanied during their training. They regarded NQNs as professionals:

‘They need to be ready and try to shift away from being err … dependent, now they need to understand they are professionals, they need to come and deliver a service err … not that they are going to be taught again, accompanied like it’s at school.’ (C, N4)

Participants’ experience of poor work readiness by NQN was related to insufficient clinical preparation during the academic programme:

‘Err the ones that I work with they are more in theory than practical; they are slow to catch, we have to teach them over and over again the practical side.’ (H, N5)

Participants experienced working with NQNs and skilling them as starting from the beginning:

‘Even practice wise there’s a lot of practice needed for the 4 years. So, when they come to the ward for practical, it seems like we start from the start, there’s a lot of work which must be …done with them, a lot of things they must be taught especially the practical side.’ (H, N9)

And she supported her response with the following experience:

‘So … once … I had one, she was a qualified sister doing … eh … comserve [*community service*] but she said in her experience or during her course she never saw blood transfused to a patient in that four years. Yeah, so I wondered in four years can you really miss blood transfusion for that four … from first year, second year, third year and fourth year … it was very much impossible for me.’ (H, N9)

The experience of some participants who had been trained under the hospital-based model made them to question the relevancy of the current nursing programme. They compared the hospital-based model with the current academic model:

‘They cannot stand on their own. In our time, we were able to work alone even if you were a staff nurse you were able to manage the ward. But with them they won’t they can’t manage the ward. They are not ready I don’t know maybe during training there is something that is not going on because after qualifying they must be able to manage the ward. They need comserve [*community service*] because after qualifying they still need guidance.’

#### Positive experience

Not all participants experienced the NQNs situation as doom and gloom. Some professional nurses experienced NQNs to have potentials. They acknowledged the individuality of NQNs and appreciated their presence in the service.

Regarding work readiness, these were their responses:

‘I think it differs from individual to individual. There are those who are keen to learn and there are those, I don’t know … it’s … it’s … it’s like you putting more pressure on them.’ (CHC, N4)

‘Like all newly appointed nurses all they needed is proper orientation with minimal supervision and some time to adjust so [*that*] they can be well grounded.’ (C, N2)

‘At the beginning each and every one needs help there and there.’ (H, N1)

Some participants commented that according to their experience, correct placement during community service add value to NQNs’ performance:

‘I think their placement is very important, where they feel like they literally want to be because if a person isn’t placed where they want to be, they are not going to perform.’ (CHC, N5)

In this theme, participants expressed how they experienced NQNs’ readiness to work and related it to NQNs not being well prepared and lacking confidence. Participants further questioned the relevancy of the current programme and the appropriateness of the curriculum. However, other participants saw potentials in NQNs and believed that correct placement would enhance their confidence.

#### Sub-theme 1.2: Dealing with individual behaviour of newly qualified nurses

Participants voiced their experience regarding the challenging behaviours and characters that the NQNs displayed. They explained that the NQNs displayed a poor work ethic shown through lack of commitment, such as idleness, neglect of their responsibilities and using cell phones most of the time.

Participants highlighted some of their experiences regarding unprofessional behaviours. They related their experiences as follows:

‘They don’t show commitment.’ (C, N1)

‘They are not professional and then they are forever on their phones like children, they just use it in front of the patients.’ (H, N5)

Some participants associated the experience of unprofessional behaviour with NQNs’ attitude:

‘Jahh … and then I can mention their attitude, they … are not really initiative or interested most of the time you have to push, do this, even if you have taught them.’ (H, N5)

‘When you show them eish … others they are those that they will show that they know better than you but at the end they come back to you to ask for some information. Like now I am having somebody who is better than me.’ (H, N2)

Despite the reports on the unprofessional behaviour, few of the participants highlighted the good attitude they experienced with some NQNs. They acknowledged NQNs’ good behaviour and were happy to interact with them:

‘Most of them are willing to learn. Some will even come and ask you how do you do this?’ (C, N3)

‘No, their attitude is not bad. They really show that they are keen to learn. They are not difficult to supervise. I have never encountered problems with them …’ (H, N7)

In this theme, participants reported about their experience of having to work with NQNs who displayed behaviour that was not professional and gave examples of such behaviours. On the other hand, other participants were satisfied with the behaviours of NQNs.

### Theme 2: Experiences as professional nurses

Participants reported on how they experienced community service. They reported on how they coped with the situation and how they managed activities as they occurred. Lack of guidelines and not being empowered to manage NQNs had a bearing on their experiences.

#### Sub-theme 2.1: Lack of empowerment

Participants were not empowered to manage the NQNs. They had to deal with situations as they unfolded: participants experienced lack of prior communication to prepare them for arrival of NQNs:

‘… [*W*]e accommodate them because we are not prepared beforehand that you’ll be having a comserve, they just come and say this is your new comserve she’ll be working in your department. You just have to take in and fit in.’ (CHC, N3)

Participants’ experience of lack of structure and not knowing what is expected of them made them feel uneasy when working with NQNs:

‘With students it’s better because I’ll be asking you “what are your objectives”, then you work on that but with comserve we don’t have a structure.’ (CHC, N4)

‘The problem is that we didn’t know what was expected with this person [*referring to the newly qualified nurses*], we didn’t know the objective of that person so yourself [*referring to herself*], you are confused, do you treat her as a student or do you treat her as a prof[*essional*] nurse.’ (C1, N1)

Participants felt drained by not being formally empowered to deal with matters of having NQNs:

‘That is the challenge, because we are not trained, we are using our own experiences. I think that’s the reason why we feel that strain, is because we don’t have the knowledge.’ (H, N1)

A heavy workload and shortage of personnel created a burden and working with NQNs added to the challenge. Participants experienced having new nurses who could not work independently as causing delays in the progress of work. They expressed their frustrations as follows:

‘It does affect our patient flow because as … if you are two in a room it delays everything, instead of the service being faster because of two professional nurses it delays the progress because now you have to start all over orientating the next person, so it delays service delivery.’ (C1, N4)

Participants expressed their experience of frustration as follows:

‘… [Y]ou know, expect them to know, 1, 2, 3 and you find out that it’s not like that, and as a sister [*referring to professional nurse*] it frustrates you [*be*]cause you look at the queue and you have two comserves to demonstrate procedures to, it is frustrating you know [*be*]cause it is time consuming.’ (CHC, N4)

Participants wished to be trained as mentors to the NQNs and their work load to be considered, when they are allocated NQNs. The presence of NQNs delayed their work progress.

#### Sub-theme 2.2: Professional nurses were being supportive

Despite the challenges that emerged in the previous themes, participants supported and guided NQNs:

‘I think err … sometimes it’s strenuous but most of the time we are there to help them so that they must develop, so that they must have a good knowledge and tomorrow they must give quality nursing care that we want.’ (H, N1)

‘I orientate them with the services I’m doing. They were willing to work as long as you orientate them in the services in … in … EPI [*Extended Programme of Immunisation*] and IMCI [*Integrated Management of Childhood Illnesses*] they are in the same room. You tell them that in the mornings: we are going to pair them and start with checking the temperature.’ (C, N2)

Participants were willing to work with NQNs and to improve their skills so that they (NQNs) can provide the needed care.

## Discussion

The experience of professional nurses’ working with NQNs revealed two major themes, which were mainly about the NQNs and themselves. Participants reported about their negative and positive experiences. Challenges that were related to the NQNs and those related to the workplace were highlighted. Despite all the challenges they were faced with, professional nurses were willing to assist the NQNs.

Participants in this study experienced NQNs as not being able to work independently and competently after qualifying from nursing education institutions. They were experienced as not being ready to work. Edward et al. (2017:327) defined work readiness as the extent to which newly registered nurses are perceived to possess the necessary knowledge and skills to work independently. Whilst Hansen-Salie and Martin (2014:540) defined it as the ability of NQNs to integrate what they have learnt in theory into practice in order to render quality patient care. Participants commented on the NQNs being more theoretical as compared with the practise. They related the NQNs’ poor work readiness to insufficient clinical preparation during the academic programme because they felt that they had to start from the beginning with the practical skills.

Work readiness has been addressed by many researchers in South Africa (Lekhuleni, Khoza & Amusa 2014:384; Tsotetsi 2012:3) and internationally (Güner 2014:845; Ulrich et al. 2010:364), as a concern for nursing practice. Edward et al. (2017:332) in their review of newly registered nurses in the United Kingdom alluded that the work readiness of NQNs continued to be a topic of debate and discussion amongst nursing professionals. Research by Alrasheeday (2019:193) on the newly graduated nurses in Saudi Arabia revealed that new nurses disclosed that they felt insufficiently prepared for clinical practice. Strauss et al. (2016:422) also cited that having new nursing graduates who are not sufficiently prepared to enter the nursing workforce is a source of concern for educators and employers, especially because the recruitment and retention of registered nurses is a serious global human resources issue. Such a global concern cannot be ignored; hence, programmes such as community service are needed to build the confidence of NQNs.

Nursing is a practical discipline that should develop students’ psychomotor, cognitive and affective skills (Netshisaulu & Maputle 2018:1); therefore, participants’ concerns with their experience of NQNs not being able to work independently and competently are significant to the nursing practice. It is assumed that when hands are more, work should go faster, which was not the case when working with NQNs who are placed for community service. Slow progress was what was happening when having NQNs in the service. When NQNs are allocated to clinical settings, the expectation is that they will implement what they have learnt and be a helping hand to the already short staffed nursing personnel; however, this seems not to be the case as working with NQNs was experienced as delaying the progress of work, especially in the Community Health Care facility and in the clinic. The delay affected the patients’ waiting time, which is constantly monitored by the District Health Department.

Participants experienced the individual unprofessional behaviours displayed by NQNs in the workplace. Newly qualified nurses were described as displaying poor work commitment because they spend time on their cell phones whilst leaving patients unattended. The behaviour frustrated the participants as they expected the NQNs to bridge the gap regarding shortage of nursing personnel. Their feelings is similar to the participants in Netshisaulu and Maputle’s (2018:5) research on ‘Expected clinical competence from midwifery graduates during community placement in Limpopo’; they expressed their disappointment at the NQNs who showed no commitment to work. Similar findings were reported in Australia by Freeling and Parker (2015:42) that experienced nurses raised concern about attitudes and ward culture of new graduate nurses. If participants experienced NQNs not to be competent and lacking confidence, it is appropriate that they should expect NQNs to show interest and commitment towards their development.

Contrary to the reports on unprofessional behaviour, some participants in this study commended the NQNs’ attitude and stated that they displayed willingness to learn. Participants acknowledged the individuality of NQNs regarding their practical skills and behaviour and attitude and recognised them as positive. Some researchers reported the behavioural complaint about professional nurses, by NQNs, citing that they found themselves in a hostile environment, where they were disrespected by the hospital staff, including the junior category of nurses, such as nursing assistants and senior staff members, including hospital managers (Nkoane & Mavhandu-Mudzusi 2020:10).

Participants in the study made it clear that there was no formal training or any form of preparation to equip them to be able to support NQNs. They assumed the role of supervising and mentoring NQNs even though they were not empowered for such responsibility. The lack of proper structure and not being prepared frustrated professional nurses as they had to deal with the situation as it unfolded. They express their experience as emotionally draining.

In a study by Panzavecchia and Pearce (2014:1121) in the United Kingdom, similar experiences were shared by participants reporting that they were not prepared to take on the role of a preceptor. As effective mentoring of NQNs required appropriate training, participants would appreciate some form of mentorship training so that they can handle the situation better. Mentoring involves the accompaniment of colleagues in areas that they are not conversant with by an expert in that area. According to Megginson and Clutterbuck in Ndaba (2013:46), a mentor is someone who has accumulated sufficient work-related experience and is able to effectively share some of this learning and experience with others. Participants wanted to be trained so that they can effectively share their accumulated experience in nursing.

Training professional nurses as mentors will benefit both professional nurses and NQNs. The same views are supported by Khunou (2018:239), who echoed that mentoring relationships can benefit both mentors and the mentees. Participants in a study by Valizadeh et al. (2016:94) stated that having appropriate mentorship training would yield effective results. Mentors in Govender, Brysiewicz and Bhengu’s (2016:68) study expressed their experience in mentoring as satisfying and an opportunity to set good behaviour as role models. They also developed confidence in working with NQNs and got positive feedback from NQNs. Spiva et al. (2013:31) recommended that professional nurses should attend workshops to prepare them for their mentoring role. Van Rooyen et al. (2018:35) alluded that being trained as a mentor provided professional nurses with opportunities to develop an assessment tools to assess NQNs, and such activities stimulated feelings of adding value to the organisation and commitment to the profession. The effectiveness and success of developing NQNs depend on the quality of mentors in clinical settings (Dinc 2014:174).

A lack of professional support and supervision has been identified by several researchers. Participants expressed their supportive behaviour towards NQNs. Despite being faced with challenges related to shortage of nursing personnel, unprofessional behaviours of NQNs and the delayed progress, participants were doing enough to support NQNs. However, other studies (Roziers et al. 2014:98 found that professional nurses were reluctant to acknowledge NQNs’ ‘pre-registration’ status and were not committed to their supervisory role as indicated in some of those studies (Lekhuleni, Khoza & Amusa 2014:388; Nkoane 2015:43): ‘NQPNs felt that they were not supported in terms of interaction in areas of employment’ (Lekhuleni et al. 2014:391). Managers and supervisors were perceived as unable to provide the necessary support to NQNs (Mammbona & Mavhandu-Mudzusi 2018:143).

On the contrary, some studies acknowledged the support that professional nurses provided to NQNs during community service. In a study by Zaayman (2016:61), NQNs verbalised their personal and professional growth and development, as they felt supported and cushioned in the hospital system, and in Govender, Brysiewicz and Bhengu’s (2015:5) study, NQNs felt supported by senior colleagues and were helped to develop confidence by receiving feedback and encouragement. Newly qualified nurses needed support, approval and supervision until they gain independence (Makua 2016:20). ‘It is of utmost importance for professional nurses to supervise, mentor and monitor NQNs for the purpose of enhancing their professional duties’ (Thopola et al. 2013:174). In this study, the participants accepted the supervisory role of NQNs. Failing to provide support might, in the long run, compromise patient care. According to Beyers (2013:51), providing support to NQNs is demonstrated as a crucial matter for their successful placement.

## Conclusion

The main aim of community service was to ensure that NQNs are supported, supervised and skilled to work independently. To achieve this objective, professional nurses are expected to provide the necessary support, whilst their key responsibility is providing quality care to patients. This is done despite the challenges related to shortages of nursing personnel, lack of structure to support and supervise NQNs and delay in the work progress caused by working with NQNs. Regardless of the fact that they were not prepared or trained as mentors to NQNs, it was apparent from participants’ experiences that they welcomed and supported NQNs during community service placement.

### Recommendations

Regular seminars and workshops should be conducted to enlighten professional nurses about the objectives of community service so that they can cooperate in realising the objectives of community service. Having them empowered in this area will make them not to expect the NQNs to be competent immediately after graduating.

The effectiveness and success of community service programme depend on the quality of mentors in clinical settings; therefore, the use of trained mentors to support NQNs during their community service placement will be beneficial. Training some professional nurses as mentors will be appropriate.

There should be collaborations between nursing education institutions and health facilities in ensuring an effective community service placement. Collaboration will assist the nursing education institutions to identify areas that need to be improved regarding nursing practice.

Nursing managers should establish a formal system such as meetings and evaluation forms for professional nurses and NQNs to share their views and air their grievances. Such a platform will ensure that professional nurses’ and NQNs’ experiences are considered.

### Limitations

The following serves as limitations for this study:

- A shortage of nursing personnel, especially in community health centres and in the clinic, limited the number of participants and saturation per institution could not be ensured except in the hospital.
- Most community health centres and the clinic had more nurses with less than five years of experience in the service, limiting the number of professional nurses available for interviews.
- The study was contextual and therefore the study findings cannot be generalised to all public health settings.

Unique contribution of this study:

- The study has documented the experiences of professional nurses working with NQNs. It therefore highlighted the need to engage professional nurses in planning the objectives of community service placement so that their experiences can contribute in reviewing the community service placement to benefit both the NQNs and professional nurses.
- The study highlighted the support needed by professional nurses to effectively provide NQNs with nursing skills, such as practical skills and physical, mental, social and emotional aspects of care that cannot be easily learnt in a class.
- Policymakers will consider the delays reported by professional nurses in working with NQN in deciding on the waiting time for patients and not be too strict in expecting reduced waiting time, especially in the community health centres and in the clinic.
- Further research can be conducted on how nurse managers can contribute in facilitating the implementation of objectives of community service.
